# Individualized concurrent chemotherapy by pretreatment plasma Epstein‐Barr viral DNA in II‐III stage nasopharyngeal carcinoma: A propensity score matching analysis using a large cohort

**DOI:** 10.1002/cam4.2343

**Published:** 2019-06-18

**Authors:** Xue‐Song Sun, Wen‐Hui Chen, Sai‐Lan Liu, Yu‐Jing Liang, Qiu‐Yan Chen, Shan‐Shan Guo, Yue‐Feng Wen, Li‐Ting Liu, Hao‐Jun Xie, Qing‐Nan Tang, Xiao‐Yun Li, Jin‐Jie Yan, Hai‐Qiang Mai, Lin‐Quan Tang

**Affiliations:** ^1^ Sun Yat‐sen University Cancer Centre Guangzhou P. R. China; ^2^ State Key Laboratory of Oncology in South China Guangzhou P. R. China; ^3^ Collaborative Innovation Centre for Cancer Medicine Guangzhou P. R. China; ^4^ Department of Nasopharyngeal Carcinoma Sun Yat‐sen University Cancer Centre Guangzhou P. R. China; ^5^ Department of Oncology The First Affiliated Hospital, Jinan University Guangzhou China

**Keywords:** chemotherapy, Epstein‐Barr virus, nasopharyngeal carcinoma, overall survival

## Abstract

**Object:**

To ascertain the treatment effect of concurrent chemotherapy (CCT) in stage II‐III nasopharyngeal carcinoma (NPC) patients with different Epstein‐Barr virus (EBV) DNA level in intensity‐modulated radiotherapy (IMRT) era.

**Methods:**

A total of 2742 patients diagnosed with stage II‐III NPC were involved in this study. Patients received IMRT with/without CCT. Overall survival (OS) was the primary endpoint. Receiver operating characteristics curve was used to determine the cut‐off value of pre‐DNA based on OS. After propensity score matching, the role of CCT was explored in patients with different EBV DNA level.

**Results:**

In our cohort, the cut‐off value of pre EBV DNA was 1460 copies/mL (area under curve [AUC], 0.695‐0.769; sensitivity, 0.766; specificity, 0.599). Patients with high EBV DNA level showed poor survival in OS, progression free survival (PFS), locoregional relapse‐free survival (LRFS) and distant metastasis‐free survival (DMFS). In patients with EBV DNA level >1460 copies/mL, the concurrent chemoradiotherapy (CCRT) group achieved higher 3‐year OS compared with IMRT groups. However, the CCRT and IMRT groups showed comparable OS in patients with EBV DNA ≤1460 copies/mL. In multivariate analyses, CCT was a protective factor for OS, PFS, and LRFS in high‐risk patients (EBV DNA level >1460 copies/mL), while not an independent prognostic factor among the low‐risk patients (EBV DNA level ≤1460 copies/mL).

**Conclusion:**

Pre‐EBV DNA could be a useful tool to guide individualized treatment for stage II‐III NPC patients. Additional CCT to IMRT improved the survival for patients with high pre‐EBV DNA, while those with low pre‐EBV DNA could not.

## 1 INTRODUCTION

Nasopharyngeal cancer (NPC) is an uncommon cancer, with an estimated 86,700 new cases in 2012, accounting for 0.6% of all cancers in China.^1^ However, it has a high incidence in the Guangdong Province, Fujian Province, and Hong Kong.^2^, ^3^ In endemic areas, the nonkeratinising undifferentiated NPC subtype comprises ~95% of cases and is inevitably correlated with Epstein‐Barr virus (EBV) infection.^4^

Radiotherapy (RT) is the only curative treatment for NPC because of its radiosensitivity.^5^ In two‐dimensional radiotherapy (2DRT) era, several studies demonstrated that concurrent chemoradiotherapy (CCRT) was recommended for locoregional advanced NPC.^6^, ^7^, ^8^ Recently, with the development of economics, mathematics, and computer science, intensity‐modulated radiotherapy (IMRT) replaced 2DRT in centers where this radiation technology was available. IMRT was superior to 2DRT in terms of locoregional control of cancer and it improved the patient's quality of life as a result of its spatial dose distribution in the target volume.^9^, ^10^, ^11^ Thus, the role of CCRT needed to be further verified in IMRT era. As the application of CCRT led to a higher incidence of late toxicities compared with RT alone,^12^ the individualized treatment was necessary according to different risk levels. Stratified by TNM staging system, previous study verified that the combination of concurrent chemotherapy (CCT) did not result in a survival benefit for stage II and low‐risk stage III NPC patients.^13^ Plasma EBV DNA levels was proven to be an important biomarker, which could monitor and predict the survival of NPC.^14^, ^15^ Our previous study established an effective prognostic nomogram with EBV DNA, which provided significantly better discrimination than TNM stage.^16^ Therefore, EBV DNA could be served as further supplement in risk stratification.

Based on these evidences, we carried out a retrospective analysis using a large cohort and long duration of follow‐up to identify the prognostic value of pre‐EBV DNA level for risk stratification among stage II‐III NPC patients and selected the candidates that might benefit from CCRT.

## 2 MATERIALS AND METHODS

### 2.1 Patients

**Figure 1:**
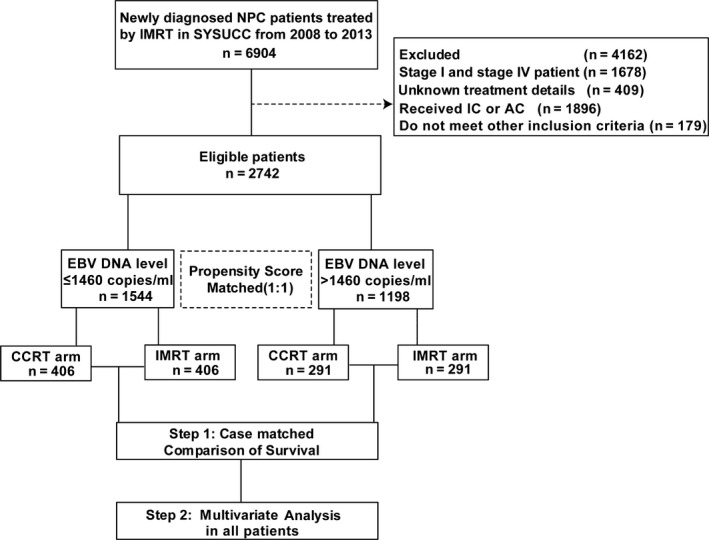
Flow chart used for patient inclusion

Between 2008 and 2013, we assessed 2742 consecutive and unselected patients with stage II‐III NPC within the Cancer Center of Sun Yat‐sen University (Guangdong, China). All patients were restaged according to the seventh TNM staging manual from the American Joint Committee on Cancer.^17^ The inclusion criteria of this study were as follows: (a) newly diagnosed stage II‐III NPC; (b) age ≥18 years; (c) Karnofsky performance score ≥70; (d) no other malignancies; (e) receiving CCRT or IMRT alone; (f) CCT was single‐agent cisplatin. Flow chart of patient inclusion was shown in Figure 1. Before the diagnosis and treatment, a sequence of evaluations was conducted, including physical examination, magnetic resonance imaging (MRI) with contrast of the nasopharynx and neck, nasopharyngoscopy, radiography of the chest or contrast‐enhanced computed tomography (CT) of the chest, abdomen ultrasound or contrast‐enhanced CT of the abdomen, electrocardiography, and bone scintigraphy. PET/CT was applied optionally. The study protocol was approved by the Research Ethics Committee of the Cancer Center of Sun Yat‐sen University.

### 2.2 Quantification of plasma EBV DNA levels

Pretreatment EBV DNA levels was measured and subjected to real‐time quantitative polymerase chain reaction (qPCR) as mentioned in previous study.^18^ The *Bam*HI‐W was the initially region of the qPCR system. The amplification primers involved W‐44F (5′‐AGTCTCTGCCTCCAGGCA‐3′), W‐119R (5′‐ACAGAGGGCCTGTCCACCG‐3′) and the dual‐labeled fluorescent probe W‐67T (5′‐[FAM]CACTGTCTGTAAAGTCCAG CCTCC[TAMRA]‐3′), which were consisted in this system. The β‐actin gene was served as a loading control, the primers 5′‐ACA GGCACCAGGGCGTGATGG‐3′ (forward), 5′‐CTCCATGTCGTCCCAGTTGGT‐3′ (reverse) and the dual labeled uorescent probe sequence 5′‐[FAM]CATCCTCACCCTGAAGTACCCCATC[TAMRA]‐3′ were applied. The cut‐off value of the EBV DNA level was defined by receiver operating characteristic (ROC) curve analysis for Overall survival (OS), which showed the best trade‐off between sensitivity and specificity.

### 2.3 Chemotherapy and RT

All patients received IMRT with or without CCT based on the treatment protocol for NPC patients at the Cancer Center of Sun Yat‐sen University. RT was performed 5 times a week at 1.8‐2.2 Gy per day by IMRT. The accumulated radiation dose to the planning target volume of a primary tumour was 66‐72 Gy. The design of the IMRT plan has been previously reported.^19^ CCT was administered using a cisplatin (80‐100 mg/m^2^, i.v.) regimen for 2‐3 cycles or dose of 30‐40 mg/m^2 ^every week during RT.

### 2.4 Outcome and follow‐up

The primary endpoint of our study was OS. Progression‐free survival (PFS), locoregional relapse‐free survival (LRFS) and distant metastasis‐free survival (DMFS) served as secondary endpoints. OS was defined as the time from the date of diagnosis to the date of death from any cause. PFS was considered the time from the date of the diagnosis to the date of first failure or death from any cause. LRFS was determined as the time from the date of the diagnosis to the date of first local and/or regional failure. DMFS was the time from the date of the diagnosis to the date of distant metastasis. After treatment, patients were evaluated every 3 months in the first 3 years and every 6 months thereafter until death. Physical examination, nasopharyngoscopy, contrast‐enhanced MRI of the nasopharynx and neck, ultrasound of the abdomen, chest radiography, and measurement of plasma levels of EBV DNA were done routinely. PET/CT was considered if necessary.

### 2.5 Statistical analyses

Statistical analyses were conducted using SPSS v23 (IBM, Armonk, IL, USA). The propensity score for each patient was calculated to estimate their probability using multivariable logistic regression models given the following covariates: age, gender, smoking, family history of NPC, T stage, N stage, EBV‐DNA level, and overall stage. Matching was carried out by the nearest neighbor‐matching method with use of a 1:1 matching protocol with a calliper of 0.05. The Pearson χ^2^ test was used to assess the statistical relationship between the subgroups. Kaplan‐Meier curves were used to compare the survival in different treatment groups with log rank test. A Cox proportional hazards regression model was used to estimate the hazard ratios (HRs) and 95% confidence intervals (CIs) in multivariate analysis. All statistical tests were 2‐tailed. *P* < 0.05 was considered statistically significant.

## 3 RESULTS

### 3.1 Patient characteristics

The baseline characteristics of the original dataset were summarized in Table 1. The entire cohort carried a male‐to‐female ratio of 2.46, and the median age was 47 years old. The median follow‐up duration was 47.5 months (range, 1.3‐90.7 months). Overall, 923 (33.7%) patients received IMRT alone and 1819 (66.3%) received CCRT. The CCRT group had a higher percentage of T3, N2, and stage III disease (*P* < 0.001), more young patients and higher EBV DNA levels (Table S1).

**Table 1:** Patient characteristics of the cohort

Characteristic	No. of patients (%)
Age, y	
≤47	1439 (52.5)
>47	1303 (47.5)
Gender	
Female	792 (28.9)
Male	1950 (71.1)
Smoking history	
No	1731 (63.1)
Yes	1011 (36.9)
NPC family history	
No	2403 (87.6)
Yes	340 (12.4)
T stage^b^	
T1	226 (8.2)
T2	866 (31.6)
T3	1650 (60.2)
N stage^b^	
N0	625 (8.2)
N1	1248 (31.6)
N2	869 (60.2)
Overall stage	
II	779 (28.4)
III	1963 (71.6)
EBV DNA level^a^	
≤1460 copies/mL	1554 (56.3)
>1460 copies/mL	1198 (43.7)
Treatment method	
IMRT alone	923 (33.7)
CCRT	1819 (66.3)

Note

Pearson χ^2^ test was used to calculate the *P*‐value.

Abbreviations: CCRT, concurrent chemoradiotherapy; EBV, Epstein‐Barr virus; IMRT, intensity‐modulated radiotherapy; NPC, nasopharyngeal carcinoma.

a The value of EBV‐DNA levels is based on receiver operating characteristic (ROC) curve analysis.

b According to the seventh edition of UICC/AJCC staging system.

### 3.2 Cut‐off value of EBV DNA level

**Figure 2:**
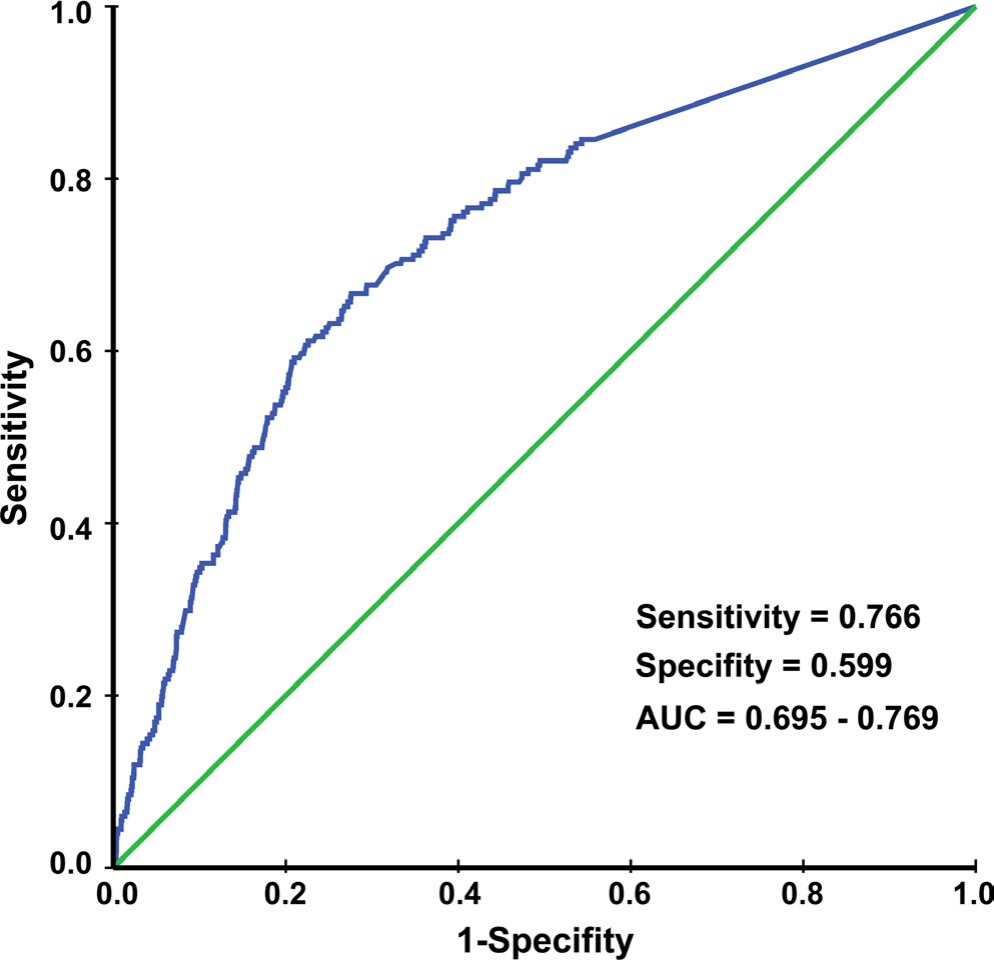
Receiver operating characteristic (ROC) curve analysis used to identify the cut‐off value of pretreatment Epstein‐Barr virus DNA

The median EBV DNA concentration for the 2742 patients was 556 copies/ml (range, 0‐44 500 000 copies/mL). Based on the ROC analysis, the cut‐off value of pre‐DNA was 1460 copies/mL for OS (sensitivity = 0.766, specificity = 0.599, 95% CI of area under curve [AUC] = 0.695‐0.769) (Figure 2). Thus, we used 1460 copies/mL as the threshold and divided patients into different risk subgroups according to EBV DNA level. We further comparatively evaluated the prognostic impact of EBV DNA. Undoubtedly, patients with EBV DNA ≤1460 copies/mL showed higher 3‐year OS and the same trend was found in PFS, LRFS, and DMFS (Figure S1). Therefore, this cut‐off value was valid, and patients with EBV DNA ≤1460 copies/mL were classified in the low‐risk group. Patients with EBV DNA >1460 copies/mL were included in the high‐risk group.

### 3.3 Multivariate analysis within the patient cohort

In the multivariate analysis of the patient cohort, the following variables were considered in the Cox proportional hazards model: age, gender, smoking, family history of NPC, T stage, N stage, level of EBV DNA, and treatment regimen. As shown in Table 2, application of CCRT was associated with a lower risk of death (HR, 0.582; 95% CI, 0.430‐0.788; *P* < 0.001), tumour progression (0.708; 0.562‐0.893; *P* = 0.004) and locoregional relapse (0.590; 0.404‐0.861; *P* = 0.006) than the application of IMRT alone. In addition, multivariate analysis revealed that the presence of high plasma EBV DNA remained an independent prognostic factor for poor OS (3.581; 2.503‐5.125; *P* < 0.001), PFS (3.353; 2.607‐4.311; *P* < 0.001), LRFS (3.049; 2.041‐4.555; *P* < 0.001) and DMFS (3.908; 2.794‐5.467; *P* < 0.001) in patients.

**Table 2:** Multivariable analysis of prognostic factors for OS, PFS, LRFS, and DMFS

Characteristic	HR (95% CI)	*P*‐value
Overall survival		
Age	1.592 (1.191‐2.130)	0.002
Gender	2.419 (1.413‐3.268)	<0.001
Smoking	1.086 (0.925‐1.275)	0.314
Family history of NPC	0.809 (0.519‐1.261)	0.349
T stage		
T2 vs T1	1.439 (0.705‐2.938)	0.317
T3 vs T1	1.945 (0.980‐3.859)	0.057
N stage		
N1 vs N0	1.033 (0.673‐1.585)	0.883
N2 vs N0	1.506 (0.967‐2.345)	0.070
EBV‐DNA level	3.581 (2.503‐5.125)	<0.001
Treatment method	0.582 (0.430‐0.788)	<0.001
Progression free survival		
Age	1.127 (0.911‐1.393)	0.270
Gender	1.543 (1.167‐2.039)	0.002
Smoking	1.025 (0.908‐1.159)	0.687
Family history of NPC	0.881 (0.638‐1.217)	0.442
T stage		
T2 vs T1	1.474 (0.889‐2.442)	0.132
T3 vs T1	1.675 (1.029‐2.727)	0.038
N stage		
N1 vs N0	1.003 (0.737‐1.364)	0.986
N2 vs N0	1.191 (0.859‐1.653)	0.295
EBV‐DNA level	3.353 (2.607‐4.311)	<0.001
Treatment method	0.708 (0.562‐0.893)	0.004
Loco‐regional relapse‐free survival		
Age	1.019 (0.718‐1.446)	0.916
Gender	1.605 (1.018‐2.531)	0.042
Smoking	0.991 (0.810‐1.213)	0.931
Family history of NPC	1.256 (0.787‐2.005)	0.340
T stage		
T2 vs T1	2.519 (0.997‐6.364)	0.051
T3 vs T1	2.028 (0.808‐5.088)	0.132
N stage		
N1 vs N0	0.941 (0.589‐1.503)	0.799
N2 vs N0	0.924 (0.547‐1.562)	0.768
EBV‐DNA level	3.049 (2.041‐4.555)	<0.001
Treatment method	0.590 (0.404‐0.861)	0.006
Distant metastasis‐free survival		
Age	1.061 (0.811‐1.387)	0.667
Gender	1.479 (1.039‐2.103)	0.030
Smoking	1.019 (0.872‐1.190)	0.817
Family history of NPC	0.844 (0.556‐1.280)	0.425
T stage		
T2 vs T1	1.064 (0.577‐1.963)	0.842
T3 vs T1	1.539 (0.866‐2.734)	0.142
N stage		
N1 vs N0	1.246 (0.806‐1.927)	0.323
N2 vs N0	1.593 (1.015‐3.500)	0.043
EBV‐DNA level	3.908 (2.794‐5.467)	<0.001
Treatment method	0.782 (0.580‐1.055)	0.107

Note

A Cox proportional hazard model was used to perform multivariate analyses. All variables were transformed into categorical variables. HRs were calculated for Age (years) (>47 vs ≤47); Gender (Male vs Female); Smoking (Yes vs No); Family history of NPC (Yes vs No); EBV DNA level (>1460 copies/mL vs ≤1460 copies/mL) and Treatment method (CCRT vs IMRT alone).

Abbreviations: CI, confidence interval; DMFS, distant metastasis‐free survival; EBV, Epstein–Barr virus; HR, hazard ratio; NPC, nasopharyngeal carcinoma; OS, Overall survival; LRFS, locoregional relapse‐free survival; PFS, progression free survival.

### 3.4 Survival outcomes within low EBV DNA group

**Figure 3:**
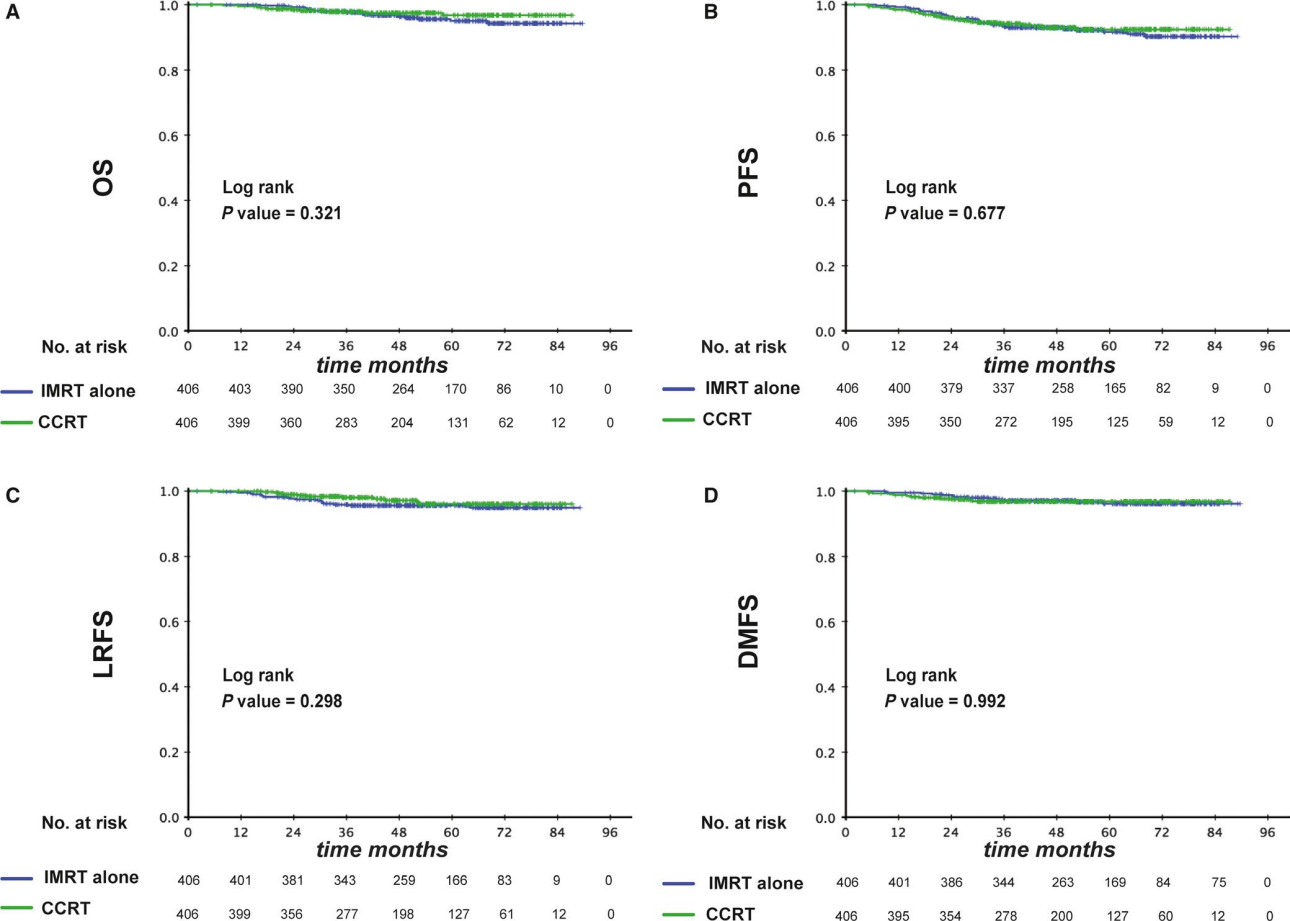
Kaplan‐Meier survival curves between the IMRT and CCRT groups in low‐risk patients (pre‐EBV DNA ≤1460 copies/mL). Shown are results for overall survival (A), progression free survival (B), locoregional relapse free survival (C), and distant metastasis free survival (D). *P* values were calculated using the unadjusted log‐rank test. EBV, Epstein–Barr virus; CCRT, concurrent chemoradiotherapy; IMRT, intensity‐modulated radiotherapy

We further evaluated the survival difference between the IMRT alone and CCRT groups among patients with low DNA. In total, 1544 patients demonstrated EBV DNA ≤1460 copies/mL. After matching, 406 pairs were selected and a well‐balanced cohort was created (Table 3). All reported parameters were balanced between the two groups, respectively, and no statistical differences were detected. The 3‐year OS, PFS, LRFS, and DMFS rates for IMRT alone vs CCRT were 97.7% vs 97.9% (*P* = 0.321), 93.3% vs 94.2% (*P* = 0.677), 95.6% vs 98.0% (*P* = 0.298) and 97.1% vs 96.9% (*P* = 0.992; Figure 3), respectively. Multivariate analysis found that there was no significant survival difference between IMRT alone and CCRT groups (*P* > 0.05 for all survival endpoints, Table 4). Therefore, IMRT alone and CCRT achieved similar outcomes in the low EBV DNA group.

**Table 3:** Clinical characteristics of patients in different risk groups according to EBV‐DNA levels

Characteristic	Low‐risk patients^a^ (n = 812)			High‐risk patients^a^ (n = 582)		
	LRRT	CCRT	*P*‐value	LRRT	CCRT	*P*‐value
Total	406	406		291	291	
Age, y						
≤47	204 (50.2)	227 (55.9)	0.122	132 (45.4)	156 (53.6)	0.056
>47	202 (49.8)	179 (44.1)		159 (54.6)	135 (46.4)	
Gender						
Female	112 (27.6)	115 (28.3)	0.876	83 (28.5)	86 (29.6)	0.855
Male	294 (72.4)	291 (71.7)		208 (71.5)	205 (70.4)	
Smoking history						
No	264 (65.0)	285 (70.2)	0.283	169 (58.1)	163 (56.0)	0.527
Yes	142 (35.0)	121 (29.8)		122 (41.9)	128 (44.0)	
NPC family history						
No	355 (87.4)	357 (87.9)	0.915	251 (86.3)	250 (85.9)	1.000
Yes	51 (12.6)	49 (12.1)		40 (13.7)	41 (14.1)	
T stage^b^						
T1	45 (11.1)	54 (13.3)	0.606	26 (8.9)	38 (13.1)	0.288
T2	167 (41.1)	159 (39.2)		94 (32.3)	88 (30.2)	
T3	194 (47.8)	193 (47.5)		171 (58.8)	165 (56.7)	
N stage^b^						
N0	152 (37.4)	158 (38.9)	0.415	42 (14.4)	36 (12.4)	0.687
N1	225 (55.4)	228 (56.2)		138 (47.4)	147 (50.5)	
N2	29 (7.1)	20 (4.9)		111 (38.1)	108 (37.1)	
Overall stage						
II	197 (48.5)	199 (49.0)	0.944	72 (24.7)	32 (25.8)	0.849
III	209 (51.5)	207 (51.0)		39 (75.3)	29 (74.2)	

Note

*P*‐value was calculated with the Pearson χ^2^ test.

Abbreviations: CCRT, concurrent chemoradiotherapy; LRRT, locoregional radiotherapy; NPC, nasopharyngeal carcinoma.

a Low‐risk patients: EBV‐DNA levels ≤1460 copies/mL; High‐risk patients: EBV‐DNA levels >1460 copies/mL.

b According to the seventh edition of UICC/AJCC staging system.

**Table 4:** Multivariate analysis of OS, PFS, LRFS, and DMFS in low‐ and high‐risk patients according to EBV DNA level

Characteristic	Low‐risk patients		High‐risk patients	
	HR (95% CI)	*P*‐value	HR (95% CI)	*P*‐value
Overall survival				
Age	3.071 (1.561‐6.042)	0.001	1.350 (0.975‐1.871)	0.071
Gender	5.398 (1.590‐18.318)	0.007	1.825 (1.160‐2.872)	0.009
Smoking	1.270 (0.924‐1.745)	0.140	1.036 (0.860‐1.248)	0.709
Family history of NPC	0.341 (0.082‐1.409)	0.137	0.955 (0.595‐1.532)	0.848
T stage				
T2 vs T1	0.911 (0.294‐2.823)	0.872	1.871 (0.732‐4.782)	0.191
T3 vs T1	1.312 (0.441‐3.901)	0.625	2.504 (1.014‐6.182)	0.047
N stage				
N1 vs N0	1.143 (0.582‐2.248)	0.698	0.990 (0.566‐1.731)	0.973
N2 vs N0	2.258 (0.936‐5.446)	0.070	1.400 (0.815‐2.406)	0.223
Treatment method	0.603 (0.307‐1.181)	0.140	0.575 (0.408‐0.811)	0.002
Progression free survival				
Age	1.211 (0.806‐1.819)	0.356	1.101 (0.858‐1.413)	0.448
Gender	2.305 (1.304‐4.074)	0.004	1.339 (0.970‐1.850)	0.076
Smoking	1.021 (0.816‐1.276)	0.858	1.039 (0.898‐1.202)	0.609
Family history of NPC	0.701 (0.353‐1.391)	0.310	0.935 (0.648‐1.350)	0.720
T stage				
T2 vs T1	1.214 (0.497‐2.969)	0.670	1.623 (0.880‐2.995)	0.121
T3 vs T1	1.960 (0.825‐4.655)	0.127	1.583 (0.877‐2.858)	0.127
N stage				
N1 vs N0	0.995 (0.633‐1.565)	0.983	1.002 (0.654‐1.538)	0.991
N2 vs N0	1.293 (0.712‐2.348)	0.399	1.163 (0.762‐1.774)	0.484
Treatment method	0.763 (0.484‐1.201)	0.242	0.672 (0.513‐0.880)	0.004
Loco‐regional relapse‐free survival				
Age	0.866 (0.463‐1.618)	0.651	1.080 (0.705‐1.652)	0.724
Gender	1.523 (0.683‐3.396)	0.304	1.655 (0.951‐2.880)	0.074
Smoking	1.093 (0.770‐1.552)	0.618	0.954 (0.746‐1.221)	0.709
Family history of NPC	0.713 (0.254‐1.999)	0.520	1.475 (0.866‐2.515)	0.153
T stage				
T2 vs T1	1.287 (0.363‐4.569)	0.696	4.489 (1.075‐18.737)	0.039
T3 vs T1	1.781 (0.508‐6.236)	0.367	2.838 (0.685‐11.763)	0.150
N stage				
N1 vs N0	1.158 (0.590‐2.272)	0.669	0.740 (0.391‐1.401)	0.355
N2 vs N0	0.875 (0.301‐2.544)	0.806	0.787 (0.416‐1.487)	0.461
Treatment method	0.526 (0.263‐1.051)	0.069	0.605 (0.384‐0.954)	0.031
Distant metastasis‐free survival				
Age	1.252 (0.706‐2.218)	0.442	1.019 (0.751‐1.382)	0.904
Gender	2.647 (1.197‐5.856)	0.016	1.243 (0.835‐1.851)	0.284
Smoking	0.878 (0.637‐1.210)	0.425	1.080 (0.903‐1.291)	0.402
Family history of NPC	0.787 (0.312‐1.984)	0.611	0.855 (0.536‐1.365)	0.512
T stage				
T2 vs T1	0.683 (0.212‐2.203)	0.524	1.232 (0.598‐2.538)	0.572
T3 vs T1	1.596 (0.546‐4.661)	0.393	1.507 (0.761‐2.981)	0.239
N stage				
N1 vs N0	1.028 (0.526‐2.006)	0.937	1.397 (0.764‐2.552)	0.277
N2 vs N0	1.777 (0.808‐3.904)	0.153	1.654 (0.912‐3.001)	0.098
Treatment method	0.870 (0.455‐1.665)	0.674	0.739 (0.528‐1.035)	0.079

Note

A Cox proportional hazard model was used to perform multivariate analyses. All variables were transformed into categorical variables. HRs were calculated for Age (years) (>47 vs ≤47); Gender (Male vs Female); Smoking (Yes vs No); Family history of NPC (Yes vs No); EBV DNA level (>1460 copies/mL vs ≤1460 copies/mL) and Treatment method (CCRT vs IMRT alone).

Abbreviations: CI, confidence interval; DMFS, distant metastasis‐free survival; EBV, Epstein–Barr virus; HR, hazard ratio; NPC, nasopharyngeal carcinoma; OS, Overall survival; LRFS, locoregional relapse‐free survival; PFS, progression free survival.

### 3.5 Survival outcomes within high EBV DNA group

**Figure 4:**
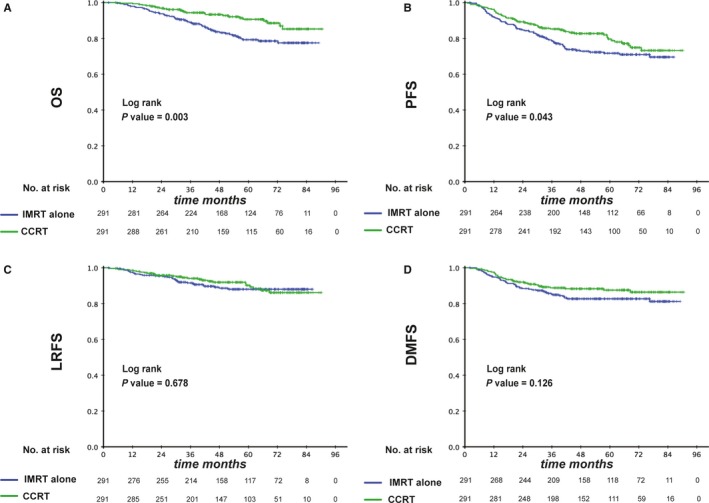
Kaplan‐Meier survival curves between IMRT and CCRT groups in high‐risk patients (pre‐EBV DNA >1460 copies/mL). Shown are the results for overall survival (A), progression free survival (B), locoregional relapse free survival (C), and distant metastasis free survival (D). *P* values were calculated using the unadjusted log‐rank test. EBV, Epstein–Barr virus; CCRT, concurrent chemoradiotherapy; IMRT, intensity‐modulated radiotherapy

Among the patients with EBV DNA >1460 copies/mL, 291 pairs were selected by PSM and baseline characteristics are presented in Table 3. The 3‐year OS, PFS, LRFS, and DMFS rates for IMRT alone vs CCRT were 88.5% vs 94.3% (*P* = 0.003), 78.3% vs 85.5% (*P* = 0.043), 91.1% vs 94.0% (*P* = 0.678) and 84.9% vs 88.8% (*P* = 0.126; Figure 4), respectively. When entered into multivariate analysis, treatment (IMRT alone vs CCRT) was identified as an independent prognostic factor for OS (HR, 0.575; 95% CI, 0.408‐0.811; *P* = 0.002), PFS (HR, 0.672; 95% CI, 0.513‐0.880; *P* = 0.004) and LRFS (HR, 0.605; 95% CI, 0.384‐0.954; *P* = 0.031; Table 3). Thus, CCRT was superior to IMRT alone among patients with high levels of EBV DNA.

## 4 DISCUSSION

To our knowledge, this is the first study to explore the role of CCT among stage II‐III NPC patients based on the pre‐EBV DNA level with a large cohort. A total of 2742 NPC patients were involved in this study. We eliminated the patients with stage I and stage IV for several reasons. Patients with stage I NPC showed low tumour burden and were treated with RT alone based on the principle of NCCN guideline.^20^ However, stage IV NPC was considered to be an advanced malignancy, which exhibited a higher tumour burden with a high‐risk of treatment failure. Thus, almost all patients received chemotherapy if the condition was tolerable. Besides, a considerable number of stage IV patients received induction chemotherapy to reduce the tumor size before RT, which was the optimum treatment model for these patients.^21^ We found that EBV DNA was an important biomarker to predict prognosis for the patients with stage II‐III, which could be used to make risk stratification. Furthermore, patients with low pre‐EBV DNA levels (≤1460 copies/ml) could not benefit from CCT, while the application of CCT could further improve the OS among high‐risk patients (pre‐EBV DNA level >1460 copies/mL).

CCRT was established as a standard treatment protocol in patients with locoregional advanced NPC because of the high‐risk of locoregional recurrence and distant metastasis.^6^, ^7^, ^8^ Among stage II NPC, we demonstrated in a phase‐III randomized study that CCT significantly improved the survival of patients with stage‐II NPC.^22^ Thus, addition of CCT during the RT period in patients with stage II‐III NPC seemed reasonable. However, all patients involved in the above studies received 2DRT. IMRT gradually replaced 2DRT. Compared with 2DRT, IMRT had the advantage of a higher dose delivery to the tumor area, decreasing the dose exposure of the normal organ.^23^, ^24^ As a result, a better locoregional control could be achieved.^9^, ^10^, ^11^

Several studies compared the survival of RT and RT plus CCT among NPC patients during the IMRT era.^13^, ^25^, ^26^ Tham et al evaluated 107 patients with stage‐IIb NPC and found no significant difference in survival between patients who underwent or did not undergo CCT.^25^ Zhang et al enrolled 661 low‐risk patients (T1N1M0, T2N0‐1M0 or T3N0M0) and similarly showed that patients receiving IMRT did not benefit from CCT.^13^ Conversely, Sun et al compared the efficacy of different treatment methods in advanced N‐stage (N2 and N3) and demonstrated that patients in the CCRT group achieved a higher 5‐OS rate compared with patients in the IMRT group.^27^ These studies proved that the application of CCT should be individualized according to the different tumor burdens.

Previous studies showed that patients with higher pre‐EBV DNA levels displayed poorer survival outcomes.^14^, ^15^ Chan et al demonstrated that with a cut‐off value of 4000 copies/mL, NPC patients could be divided into a poor‐risk group and a good‐risk group.^15^ Similarly, Lin et al found that patients with high levels of EBV DNA (more than 1500 copies/mL) suffered higher disease recurrence.^14^ Our results were consistent with these two studies, with the cutoff 1460 copies/mL, which was similar to Lin's report, supporting the prognostic value of EBV DNA in stage II‐III NPC.

In the entire cohort, the pre‐EBV DNA level showed great predictive value in all survival rates, indicating that it was credible to be used to stratify risk level. In stratified analysis according to pre‐EBV DNA level, a different scenario was observed. Among staged II‐III patients with low EBV DNA, CCRT and IMRT achieved similar survival outcomes; while for those with high EBV DNA, patients that received CCT achieved higher OS. Multivariate survival analysis showed that the application of chemotherapy was a protective factor for OS, PFS, and LRFS. Reasonably, patients with high EBV DNA suffered higher tumour burden; therefore, a more intensive treatment method, such as the application of induction chemotherapy was necessary for these patients to further eliminate tumors.

As the strong prognostic value of EBV DNA, our group launched two prospective clinical trials. In patients with stage III‐IV and EBV DNA >4000 copies/mL, we explored the effect of immunotherapy using tumour infiltrating lymphocytes after CCRT (NCT 02421640). Meanwhile, in low risk patients (stage III‐IV and EBV DNA <4000 copies/mL), we compared the survival rates between patients receiving 2 or 3 cycles cisplatin based CCT (100mg/m2) (NCT 02871518). This study showed the important basis for individualized treatment in stage II‐III NPC patients according to EBV DNA level. Moreover, it provided important theoretical support for another prospective clinical trial within low risk II‐III NPC patients, which investigate the role of CCT in these patients. The 1500 copies/mL was the proper cut‐off value to select the low risk patients based on this study and clinical application.

Several limitations existed in our study. First, this is a retrospective study and the selective bias is unavoidable. We used several methods to minimize the unbalance, such as PSM analysis and multivariate analysis. Second, all patients were from an NPC‐epidemic region in one treatment center. A multicentre prospective study is needed to validate our findings.

In conclusion, our study revealed that stage II‐III patients with high pre‐EBV DNA could benefit from additional CCT along with IMRT, whereas patients with low pre‐EBV DNA could not, indicating that pre‐EBV DNA could be a useful tool to help guide individualized treatment.

## ETHICS APPROVAL AND CONSENT TO PARTICIPATE

This retrospective study was approved by the Clinical Research Committee of Sun Yat‐sen University Cancer Center. Patients were required to provide written informed consent before enrolling in the study.

## CONSENT FOR PUBLICATION

Not applicable.

## CONFLICT OF INTERESTS

The authors declare no competing interests.

### DATA AVAILABILITY STATEMENT

The datasets used and/or analyzed during this study are available from the corresponding author on reasonable request.
