# Supplementary figures and images for: Individualized concurrent chemotherapy by pretreatment plasma Epstein‐Barr viral DNA in II‐III stage nasopharyngeal carcinoma: A propensity score matching analysis using a large cohort
Source: Cancer Med. 2019 Jun 18;8(9):4214–25. doi: 10.1002/cam4.2343 (PMC6675745; doi:10.1002/cam4.2343)

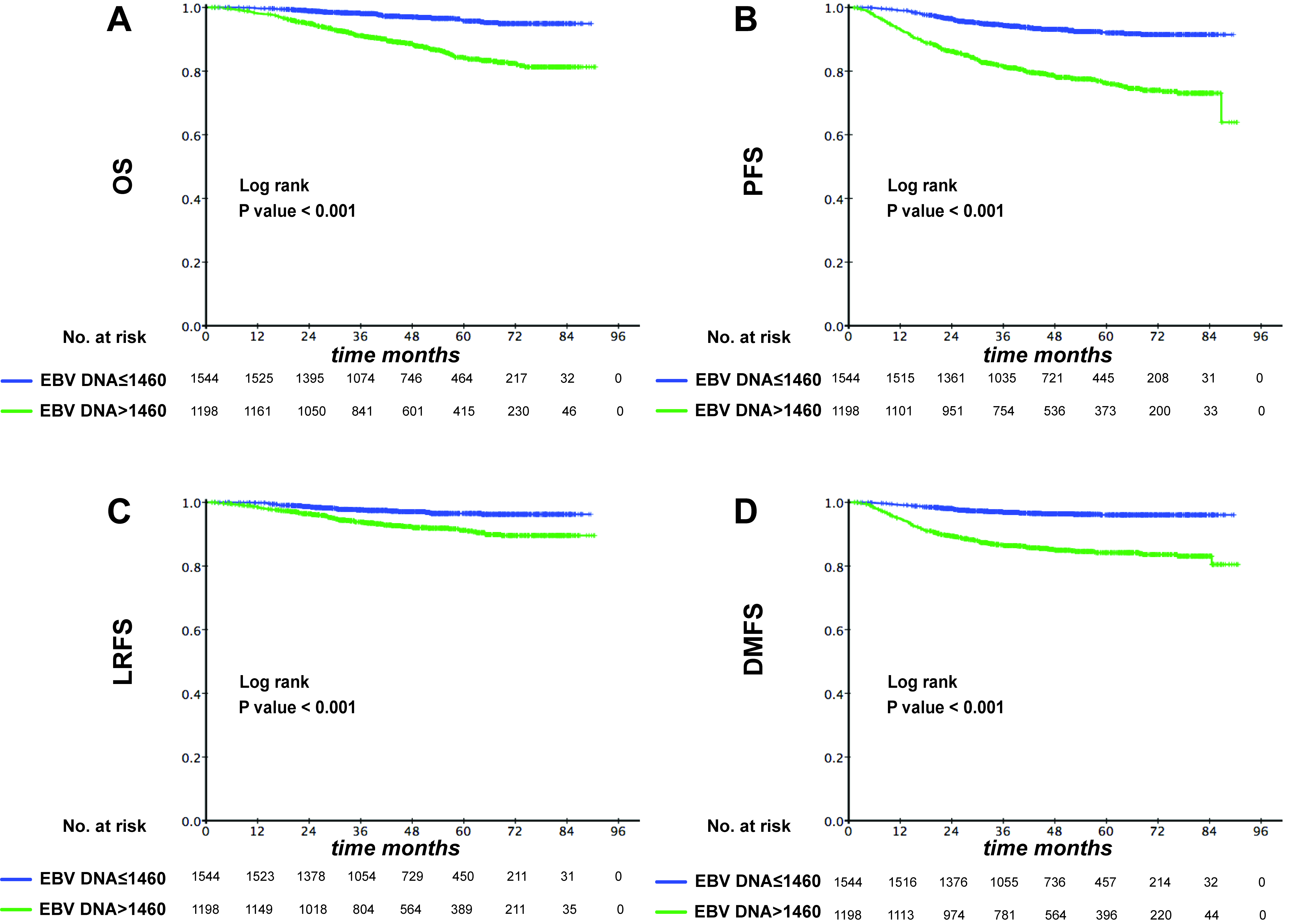

Supplement: Supplementary file 1 [file CAM4-8-4214-s001.tif]
