# Supplementary material for: Individualized concurrent chemotherapy by pretreatment plasma Epstein‐Barr viral DNA in II‐III stage nasopharyngeal carcinoma: A propensity score matching analysis using a large cohort
Source: Cancer Med. 2019 Jun 18;8(9):4214–25. doi: 10.1002/cam4.2343 (PMC6675745; doi:10.1002/cam4.2343)
Supplement: Supplementary file 2 [file CAM4-8-4214-s002.docx]

Table S1: Baseline characteristics of patients in the IMRT and CCRT group in original the cohort

| **Characteristic** | IMRT alone (n=923) | CCRT (n=1819) | *P*-value |
| --- | --- | --- | --- |
| **Total** | 923 | 1819 |  |
| **Age, y** |  |  |  |
| ≤47 | 401(43.4) | 1038(57.1) | <0.001 |
| >47 | 522(56.6) | 781(42.9) |  |
| **Gender** |  |  |  |
| Female | 284(30.8) | 508(27.9) | 0.121 |
| Male | 639(69.2) | 1311(72.1) |  |
| **Smoking history** | |  |  |
| No | 594(64.4) | 1137(62.5) | 0.343 |
| Yes | 329(35.6) | 682(37.5) |  |
| **NPC family history** | |  |  |
| No | 798(86.5) | 1604(88.2) | 0.196 |
| Yes | 125(13.5) | 215(11.8) |  |
| **T stage*** |  |  |  |
| T1 | 103(11.2) | 123(6.8) | <0.001 |
| T2 | 447(48.4) | 419(23.0) |  |
| T3 | 373(40.4) | 1277(70.2) |  |
| **N stage*** |  |  |  |
| N0 | 345(37.4) | 280(15.4) | <0.001 |
| N1 | 438(47.5) | 810(44.5) |  |
| N2 | 140(5.1) | 729(40.1) |  |
| **Overall stage** | |  |  |
| II | 487(52.8) | 292(16.1) | <0.001 |
| III | 436(47.2) | 1527(83.9) |  |
| **EBV DNA level#** | |  |  |
| ≤1460 copies/ml | 602(65.2) | 942(51.8) | <0.001 |
| >1460 copies/ml | 321(34.8) | 877(48.2) |  |

Abbreviations: HBV = hepatitis B virus; NPC = nasopharyngeal carcinoma.

# The value of EBV-DNA levels is based on receiver operating characteristic (ROC) curve analysis.

^*^According to the 7th edition of UICC/AJCC staging system.

P values were calculated by the Chi-square test.
